# Effect of acute hypoxemia on cerebral blood flow velocity control during lower body negative pressure

**DOI:** 10.14814/phy2.13594

**Published:** 2018-02-21

**Authors:** Noud van Helmond, Blair D. Johnson, Walter W. Holbein, Humphrey G. Petersen‐Jones, Ronée E. Harvey, Sushant M. Ranadive, Jill N. Barnes, Timothy B. Curry, Victor A. Convertino, Michael J. Joyner

**Affiliations:** ^1^ Department of Anesthesiology Mayo Clinic Rochester Minnesota; ^2^ Center for Research and Education in Special Environments Department of Exercise and Nutrition Sciences University at Buffalo Buffalo New York; ^3^ Nonin Medical Inc Plymouth Minnesota; ^4^ Mayo Clinic School of Medicine and Science Mayo Clinic Rochester Minnesota; ^5^ Department of Kinesiology University of Wisconsin‐Madison Madison Wisconsin; ^6^ US Army Battlefield Health & Trauma Center for Human Integrative Physiology Fort Sam Houston Texas

**Keywords:** Central hypovolemia, cerebrovascular control, hypoxemia, hypoxia, lower body negative pressure, Simulated hemorrhage

## Abstract

The ability to maintain adequate cerebral blood flow and oxygenation determines tolerance to central hypovolemia. We tested the hypothesis that acute hypoxemia during simulated blood loss in humans would cause impairments in cerebral blood flow control. Ten healthy subjects (32 ± 6 years, BMI 27 ± 2 kg·m^−2^) were exposed to stepwise lower body negative pressure (LBNP, 5 min at 0, −15, −30, and −45 mmHg) during both normoxia and hypoxia (F_i_O_2_ = 0.12–0.15 O_2_ titrated to an SaO_2_ of ~85%). Physiological responses during both protocols were expressed as absolute changes from baseline, one subject was excluded from analysis due to presyncope during the first stage of LBNP during hypoxia. LBNP induced greater reductions in mean arterial pressure during hypoxia versus normoxia (MAP, at −45 mmHg: −20 ± 3 vs. −5 ± 3 mmHg, *P* < 0.01). Despite differences in MAP, middle cerebral artery velocity responses (MCAv) were similar between protocols (*P* = 0.41) due to increased cerebrovascular conductance index (CVCi) during hypoxia (main effect, *P* = 0.04). Low frequency MAP (at −45 mmHg: 17 ± 5 vs. 0 ± 5 mmHg^2^, *P* = 0.01) and MCAv (at −45 mmHg: 4 ± 2 vs. −1 ± 1 cm·s^−2^, *P* = 0.04) spectral power density, as well as low frequency MAP‐mean MCAv transfer function gain (at −30 mmHg: 0.09 ± 0.06 vs. −0.07 ± 0.06 cm·s^−1^·mmHg^−1^, *P* = 0.04) increased more during hypoxia versus normoxia. Contrary to our hypothesis, these findings support the notion that cerebral blood flow control is not impaired during exposure to acute hypoxia and progressive central hypovolemia despite lower MAP as a result of compensated increases in cerebral conductance and flow variability.

## Introduction

Hemorrhage is one of the leading causes of accidental death (Boulanger et al. 2007) and is the leading cause of preventable death on the battlefield (Eastridge et al. 2011, 2012). Inadequate cerebral blood flow and oxygenation is the final common pathway to loss of consciousness from blood loss, and thus represents an important area of investigation. Maintenance of cerebral blood flow during hemorrhage modifies and/or challenges most of the major physiological regulatory mechanisms, including cerebral autoregulation (Lassen 1959), arterial blood gases (Ainslie and Ogoh 2010), neurovascular coupling (Rickards 2015), perfusion pressure (Rowell 1993), and cerebral autonomic vascular control (Levine and Zhang 2008). The balance of these regulatory factors ultimately determines whether cerebral blood flow is maintained or decreases under progressive blood loss.

Progressive reductions in central blood volume are not tolerated well during hypoxic conditions (Heistad and Wheeler 1970; Malhotra and Murthy 1977; Fulco et al. 1985; Rowell and Blackmon 1989; Rowell 1993; Sagawa et al. 1993; Rickards and Newman 2002; Blaber et al. 2003; Halliwill and Minson 2005). In normovolemic conditions the cerebral circulation is sensitive to variations in arterial oxygen and carbon dioxide. Acute hypoxemia causes a compensatory vasodilation which results in an increase in cerebral blood flow (Rowell 1993). However, hypoxemia also provokes a hyperventilatory response that reduces arterial carbon dioxide and causes cerebral vasoconstriction, which attenuates the hypoxemia‐induced vasodilation (Heistad and Abboud 1980). Moreover, acute hypoxemia has additional effects on the beat‐to‐beat dynamic control of cerebral blood flow in response to small changes in perfusion pressures (Nishimura et al. 2010; Iwasaki et al. 2011). In a study comparing cerebral blood flow responses in response to head up tilt under normobaric and hypobaric conditions, Blaber et al. (2003) found that baseline supine normobaric respiration rate and CO_2_ reactivity of cerebral blood flow were related to hypobaric head up tilt time to presyncope. Hypoxic syncope during head up tilt thus appears to be related to insufficient cerebral perfusion owing to cerebral vasoconstriction as a result of hypoxic hyperventilation‐induced hypocapnia. If impaired cerebrovascular control contributes to the impaired tolerance to hemorrhage and/or progressive reductions in central blood volume, it is a clinically relevant concern in the context of chest wall injuries and injuries sustained at altitude.

Lower body negative pressure (LBNP) is an established technique used to mimic the physiological responses (Helmond et al. 2015; van Helmond et al. 2016), including hemodynamic (Hinojosa‐Laborde et al. 2014, 2016; Johnson et al. 2014), and cerebral blood flow responses (Rickards et al. 2015a), to blood loss in humans. Therefore, the goal of this study was to test the hypothesis that acute hypoxemia during simulated blood loss (i.e., LBNP) in humans would cause impairments in cerebral blood flow control.

## Methods

### Ethical approval

This study was approved by the Mayo Clinic Institutional Review Board (IRB‐nr: 13‐007418) and conformed to the standards set by the 2008 revision of the Declaration of Helsinki (Williams 2008). Prior to participation, all subjects provided written informed consent after all procedures and study risks were fully explained.

### Subjects

Ten healthy men (age: 32 ± 6 years; height: 181.8 ± 6.8 cm; weight: 88.4 ± 8.8 kg; BMI: 26.7 ± 1.8 kg·m^−2^) participated in this study. Subjects were nonsmokers, and did not take any medications. All subjects reported to be free of cardiovascular, respiratory, neurologic, and metabolic disease. Subjects refrained from alcohol, caffeine, and exercise for 24 h and fasted for 12 h prior to the study visit. Upon reporting to the Clinical Research and Trials Unit (CRTU) of the Mayo Clinic at 07:00, subjects consumed a snack (Clif Bar; Shelton, CT, USA; 240 kcals) and drank 250 mL of water. Subjects were studied in the supine position in a temperature‐controlled room (20–22°C). To ensure subject safety, a board‐certified anesthesiologist was present throughout the study day.

### Experimental design

**Figure 1:**
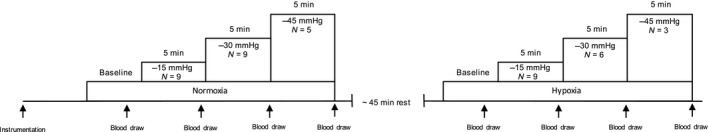
Study protocol and the number of subjects that completed each stage of LBNP during each condition.

The normoxia and hypoxia protocols were performed on the same day and Figure 1 illustrates the study protocol. We used the initial stages of the US Army Institute for Surgical Research LBNP protocol (Cooke et al. 2004) that has been validated for a wide range of physiological responses to simulate blood loss in humans (Johnson et al. 2014; Helmond et al. 2015; Rickards et al. 2015a; van Helmond et al. 2016) and primates (Hinojosa‐Laborde et al. 2014, 2016). After instrumentation for blood draws and physiological monitoring, the normoxia protocol commenced. Because we hypothesized that hypoxia would induce impaired cerebral blood flow control with consequential presyncope, we performed the normoxia protocol first. After the normoxia protocol, subjects rested quietly for 45 min in the supine position before the hypoxia protocol started. During the hypoxia protocol, the concentration of inhaled O_2_ (12–15%) was titrated to an arterial oxygen saturation of ~85% using a gas blender and nonrebreathing facemask. The protocols were terminated if mean arterial pressure fell by 30%, systolic blood pressure dropped below 80 mmHg, or the subject began to experience symptoms of presyncope or syncope. Arterial blood samples were collected at baseline and after the 5 min of each LBNP stage for the analysis of blood gases, which extended each LBNP stage by ~1 min. If a protocol was terminated early, blood samples were obtained immediately upon the decision to terminate the protocol.

### Hemodynamic measurements

A three‐lead electrocardiogram was used to continuously record heart rate (HR, Cardiocap/5, Datex‐Ohmeda, Louisville, CO). A 20‐gauge, 5‐cm catheter was placed into the brachial artery under local anesthesia (2% lidocaine) using aseptic techniques and ultrasound guidance. The catheter was attached to a high‐resolution transducer positioned at heart level to obtain continuous brachial artery blood pressure waveforms.

### Respiratory measurements

Breath‐by‐breath ventilation was measured using a turbine transducer (VacuMed, Vacumetrics Inc., Ventura, CA). Inspired and expired gases were monitored via a sample line connected to the facemask (Cardiocap/5, Datex‐Ohmeda, Louisville, CO).

### Cerebrovascular measurements

Subjects were imaged using a 2‐MHz transcranial Doppler (TCD) probe (Neurovision System, Multigon, Yonkers, NY) to estimate middle cerebral artery blood velocity (MCAv). The basal portion of the left MCA was insonated by placement of the probe over the temporal bone just above the zygomatic arch in front of the ear. The Doppler signal was optimized by variation in the sample volume depth in incremental steps and variation in the angle of insonation to obtain the best quality signal. Once the optimal signal was determined, the probe was secured with a headband device to maintain a constant angle throughout the protocol. Additionally, images of the common carotid artery (CCA) were obtained using Doppler ultrasound and 7.5–10.0 mHz linear‐array probe (Terason Echo, Teratech, Burlington, MA) to measure CCA diameter. These measurements were obtained by manually stabilizing the probe over the CCA and readjusting the probe position if subjects moved.

### Blood gases

Blood was collected from the brachial artery catheter for the measurement of arterial blood gases. Blood was collected in 3 mL syringes and analyzed using an automated blood gas analyzer (ABL‐800, Radiometer, Brea, CA) by the Department of Laboratory Medicine and Pathology and the Immunochemistry Core Laboratory of the Clinical Research and Trials Unit of the Mayo Clinic.

### Data

Data were collected at 500 Hz (Windaq, DATAQ Instruments, Akron, OH) and stored on a laboratory computer for offline analysis with signal‐processing software (WinCPRS, Absolute Aliens Oy, Turku, Finland). Physiological data were continuously monitored throughout both protocols, and data were analyzed and averaged over the last 3 min of baseline and over the last minute of each LBNP stage for statistical analysis. For spectral and transfer function analyses the last 3 min of each LBNP stage were used. The cumulative stress index was calculated as the cumulative product of LBNP pressure and time at each LBNP stage. MAP and mean MCAv were calculated as the area under the arterial pressure and MCAv curves. Analysis of CCA diameter was carried out using an automated edge‐detection software system (Medical Imaging Applications, Coralville, IA), averaging the CCA diameter over each cardiac cycle. The vertical and horizontal calibration was set based on the ultrasound settings for each individual participant. Following the calibration, the region of interest was set on the portion of the artery where the walls were most clear. Stroke volume (SV) was calculated based on the brachial arterial pressure waveform (Jellema et al. 1996). Cardiac output (CO) was derived as the product of SV and HR obtained by electrocardiogram. Cerebrovascular conductance index (CVCi) was calculated as MCAv/MAP.

Arterial pressure‐cerebral blood velocity relationships were also explored via transfer function analysis. Beat‐to‐beat time domain MAP and mean MCAv waveforms were made equidistant by linear interpolation and resampled at 5 Hz. Data were then passed through a low‐pass filter with a cut‐off frequency of 0.5 Hz. Data sets were decomposed into power spectra by fast Fourier‐transform with a Hanning window. Spectral power was expressed as the integrated area within the low‐frequency range of 0.04–0.15 Hz. We calculated the coherence between MAP and mean MCAv by dividing the squared cross‐spectral densities of the two signals by the product of the individual autospectra. Transfer function gain and phase between MAP and mean MCAv represent frequency dependence and can be used to assess dynamic cerebral blood flow‐pressure relationships (Giller 1990; Zhang et al. 1998a). Transfer function gain and phase were considered valid and averaged only when the coherence between the two spectra was ≥0.5.

### Statistical analysis

Data were analyzed with SPSS (version 22; IBM, Armonk, NY). Baseline data were evaluated by paired *t*‐test. Because we encountered several baseline differences between the hypoxia and normoxia protocols, the LBNP‐induced changes in hemodynamic, respiratory, and cerebrovascular variables were expressed and analyzed as absolute change compared to baseline. The gain between changes in MAP and mean MCAv was calculated to assess the arterial pressure‐cerebral blood flow velocity relationship. To address the potential issue of missing data due to subjects becoming presyncopal we performed the primary analysis on LBNP‐induced changes between conditions using mixed linear model analysis (Little and Rubin 2002; Rickards et al. 2015a) which have the capacity to handle unbalanced data (i.e., subjects are included in the analysis even if they do not have complete data). Mixed linear models were used to test if LBNP‐induced changes between protocols in hemodynamic, respiratory, and cerebrovascular variables were different between protocols. “Protocol” (normoxia/hypoxia) and “LBNP stage” (−15, −30, and −45 mmHg) were included as fixed factors and “Subject” was included as random factor. To supplement this analysis two‐sided paired *t*‐tests with Bonferroni correction were performed to determine at which LBNP level differences existed if a significant main or interaction effect was detected for “Protocol”. To analyze whether hypoxia influenced the absolute hemodynamic, respiratory, and cerebrovascular values at which presyncope occurred we compared absolute values during the last minute prior to presyncope between conditions using unpaired *t*‐tests. Data are presented as mean ± SE. The alpha level was set at 0.05.

## Results

**Figure 2:**
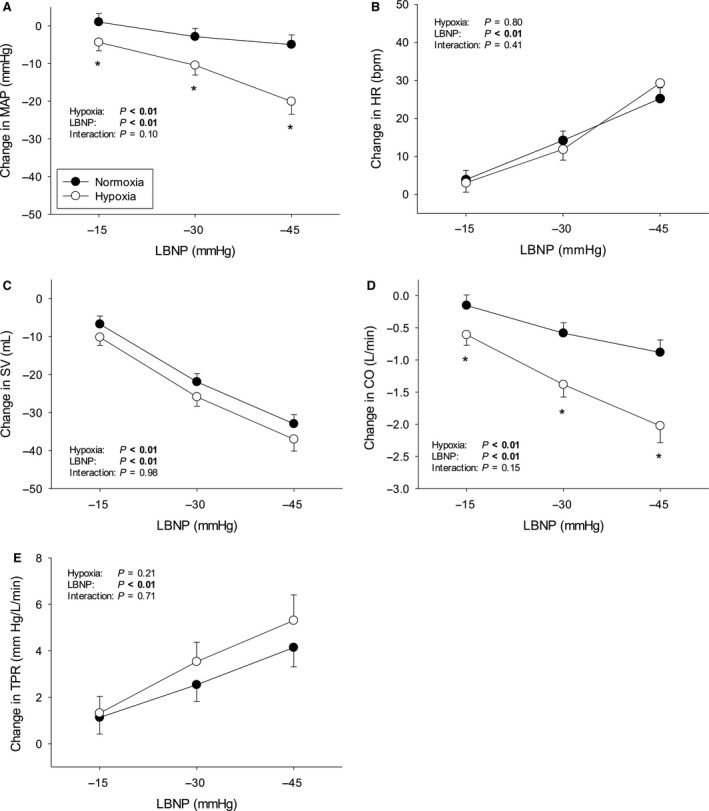
Hemodynamic responses to LBNP from baseline during normoxia and hypoxia. (A) Mean arterial pressure, MAP; (B) Heart rate, HR; (C) Stroke volume, SV; (D) Cardiac output, CO; and (E) Total peripheral resistance, TPR. LBNP = lower body negative pressure. *P ≤ 0.05 versus normoxia. Values are means ± standard error.

**Figure 3:**
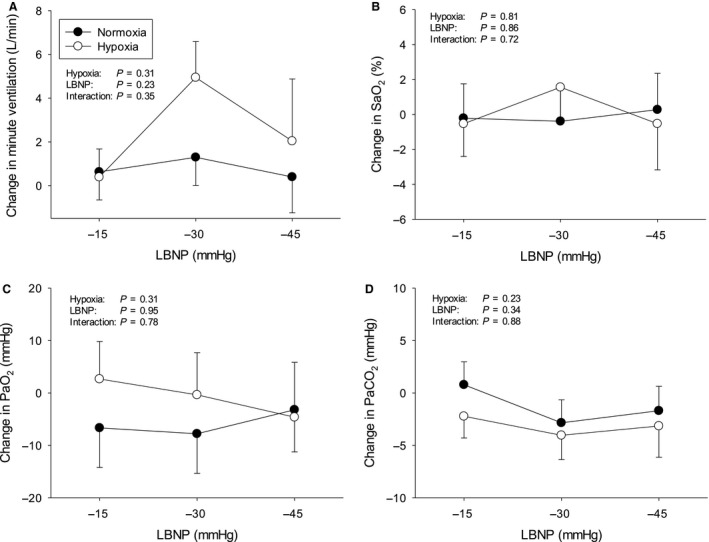
Respiratory responses to LBNP from baseline during normoxia and hypoxia. (A) Minute ventilation; (B) Arterial saturation of O_2_, SaO_2_; (C) Arterial partial pressure of O_2_, PaO_2_; and (D) Arterial partial pressure of CO_2_, PaCO_2_. LBNP = lower body negative pressure. *P ≤ 0.05 versus normoxia. Values are means ± standard error.

**Figure 4:**
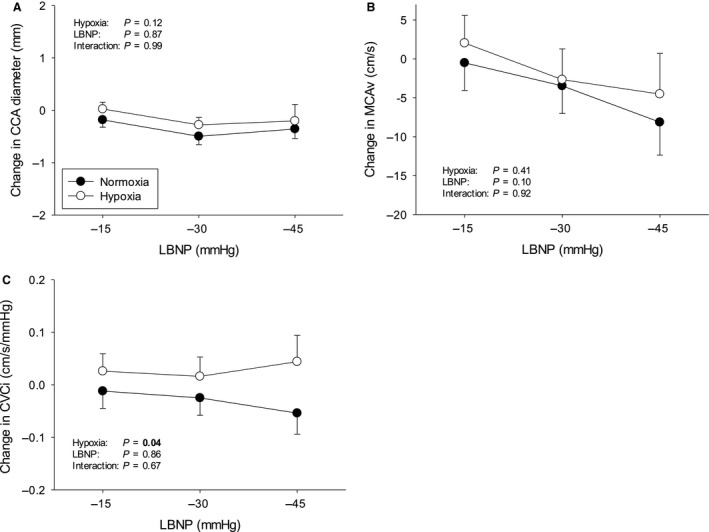
Cerebrovascular responses to LBNP from baseline during normoxia and hypoxia. (A) Common carotid artery (CCA) diameter; (B) Middle cerebral artery velocity, MCAv; and (C) Cerebral vascular conductance index, CVCi. LBNP = lower body negative pressure. *P ≤ 0.05 versus normoxia. Values are means ± standard error.

**Figure 5:**
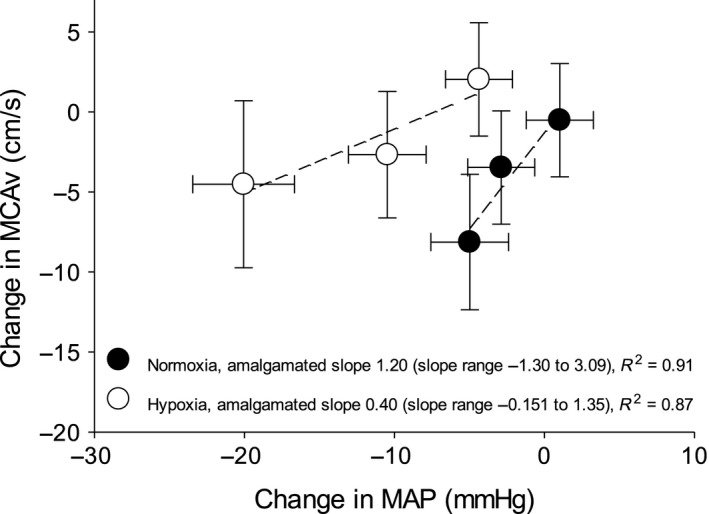
Linear regressions between amalgamated changes from baseline in MCAv and MAP during normoxia and hypoxia. MAP = mean arterial pressure; MCAv  =  middle cerebral artery velocity. Each circle represents means ± standard errors at a level of LBNP.

**Figure 6:**
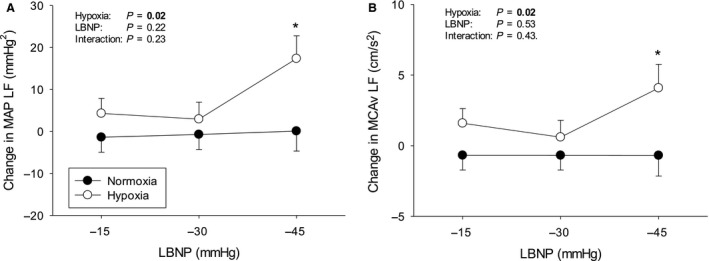
LBNP‐induced changes from baseline in spectral measures during normoxia and hypoxia. (A) Mean arterial pressure (MAP) low frequency (LF) power spectral density and (B) Mean middle cerebral artery velocity (MCAv) LF power spectral density. LBNP = lower body negative pressure. * *P* ≤ 0.05 versus normoxia. Values are means ± standard error.

**Figure 7:**
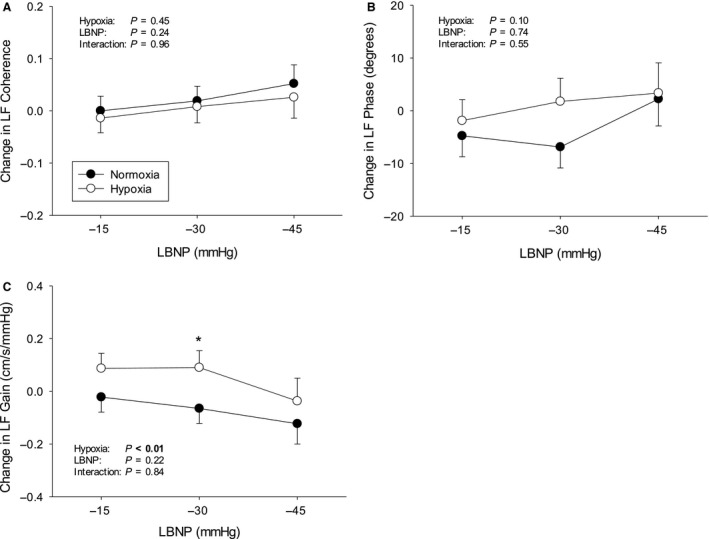
LBNP‐induced changes from baseline in dynamic cerebrovascular control measures during normoxia and hypoxia. (A) Low frequency (LF) coherence; (B) LF phase; and (C) LF gain. LBNP = lower body negative pressure. * *P* ≤ 0.05 versus normoxia. Values are means ± standard error.

Protocols were terminated early in a number of subjects due to presyncopal symptoms – Figure 1. The subject who did not complete any stage during hypoxia was excluded from analysis altogether, since our aim was to compare compensatory physiological changes in response to LBNP with hypoxemia versus normoxemia. The average cumulative stress index was lower during hypoxia (312 ± 72 vs. 476 ± 57 mmHg·min^−1^, *P* < 0.01). Data from uncompleted stages were excluded from analysis. The mean hemodynamic, respiratory, and cerebrovascular values obtained at baseline during both protocols are presented in Table 1. The LBNP‐induced changes in hemodynamic, respiratory, and cerebrovascular parameters during normoxia and hypoxia are displayed in Figures 2, 3, and 4, respectively. Regression line slopes produced from the stimulus–response relationships between the amalgamated MAP and mean MCAv responses are illustrated in Figure 5. The LBNP‐induced changes in MAP and MCAv power spectral density and transfer function measures during normoxia and hypoxia are shown in Figures 6 and 7, respectively. The absolute hemodynamic, respiratory, and cerebrovascular values just prior to presyncope during both conditions are displayed in Table 2.

**Table 1:** Baseline physiological values during normoxia and hypoxia

	Normoxia	Hypoxia	*P*‐value
*Hemodynamic variables*			
MAP, mmHg	103 ± 2	102 ± 3	0.67
HR, beats·min^−1^	61 ± 2	73 ± 3	**<0.01**
SV, mL	73 ± 4	73 ± 3	0.85
CO, L·min^−1^	4.4 ± 0.1	5.3 ± 0.3	**<0.01**
TPR, mmHg·min^−1^·mL^−1^	23 ± 1	19 ± 1	**<0.01**
*Respiratory variables*			
Respiration rate, breaths·min^−1^	12 ± 1	12 ± 1	0.96
Total ventilation, L·min^−1^	9.0 ± 0.5	10.5 ± 0.6	0.05
SaO_2_, %	97 ± 0.3	85 ± 1.5	**<0.01**
PaO_2_, mmHg	111 ± 5	52 ± 4	**<0.01**
PaCO_2_, mmHg	41 ± 1	39 ± 1	0.40
*Cerebrovascular variables*			
CCA diameter, mm	7.8 ± 0.22	7.7 ± 0.17	0.46
MCAv, cm·s^−1^	62 ± 5	56 ± 3	0.23
CVCi, cm·s^−1^·mmHg^−1^	0.61 ± 0.05	0.57 ± 0.03	0.44
*Spectral variables*			
MAP LF power, mmHg^2^	6.2 ± 2.9	4.8 ± 0.8	0.57
MCAv LF power, cm·s^−2^	1.9 ± 0.9	1.2 ± 0.3	0.39
*Dynamic autoregulation variables*			
LF coherence	0.73 ± 0.02	0.73 ± 0.02	0.88
LF phase, degrees	15 ± 4	13 ± 3	0.75
LF gain, cm·s^−1^·mmHg^−1^	0.67 ± 0.09	0.65 ± 0.07	0.31

Bold indicates significantly different values.

MAP, mean arterial pressure; HR, heart rate; SV, stroke volume; CO, cardiac output; TPR, total peripheral resistance; SaO_2_, arterial saturation of O_2_; PaO_2_, arterial partial pressure of O_2_; PaCO_2_, arterial partial pressure of CO_2_; CCA, common carotid artery; MCAv, middle cerebral artery velocity; CVCi, cerebral vascular conductance index; LF, low‐frequency.

Values are means ± standard error, *n* = 9.

**Table 2:** Physiological values during the last minute prior to presyncope

	Normoxia	Hypoxia	*P*‐value
*Hemodynamic variables*			
MAP, mmHg	86 ± 6	82 ± 6	0.71
HR, beats·min^−1^	80 ± 7	77 ± 7	0.60
SV, mL	46 ± 2	56 ± 7	0.34
CO, L·min^−1^	3.7 ± 0.4	4.2 ± 0.5	0.68
TPR, mmHg·min^−1^·mL^−1^	25 ± 2	22 ± 2	0.36
*Respiratory variables*			
Respiration rate, breaths·min^−1^	14 ± 1	13 ± 1	0.86
Total ventilation, L·min^−1^	11.7 ± 0.4	15.3 ± 3.2	0.41
SaO_2_, %	96 ± 0.3	90 ± 2	0.09
PaO_2_, mmHg	99 ± 8	67 ± 11	0.11
PaCO_2_, mmHg	40 ± 2	36 ± 2	0.21
*Cerebrovascular variables*			
CCA diameter, mm	7.1 ± 0.2	7.5 ± 0.2	0.22
MCAv, cm·s^−1^	54 ± 8	51 ± 4	0.66
CVCi, cm·s^−1^·mmHg^−1^	0.63 ± 0.05	0.57 ± 0.03	0.34

MAP, mean arterial pressure; HR, heart rate; SV, stroke volume; CO, cardiac output; TPR, total peripheral resistance; SaO_2_, arterial saturation of O_2_; PaO_2_, arterial partial pressure of O_2_; PaCO_2_, arterial partial pressure of CO_2_; CCA, common carotid artery; MCAv, middle cerebral artery velocity; CVCi, cerebral vascular conductance index.

Values are means ± standard error, *n* = 4 for normoxia, *n* = 6 for hypoxia.

### Effect of hypoxia on hemodynamic responses to LBNP

At baseline, TPR was lower during hypoxia (*P* < 0.01), whereas HR (*P* < 0.01) and CO (*P* < 0.01) were higher (Table 1). LBNP evoked pronounced hemodynamic changes from baseline during both normoxia and hypoxia (Fig. 2). MAP, SV, and CO decreased more under hypoxic conditions (<0.01 for all), while HR and TPR increased similarly (*P* > 0.05 for both).

### Effect of hypoxia on respiratory responses to LBNP

Baseline SaO_2_ (*P* < 0.01) and PaO_2_ (*P* < 0.01) were lower during hypoxia (Table 1). Minute ventilation increased similarly during LBNP between protocols and there was large inter‐subject variability in the ventilatory response (Fig. 3). LBNP‐induced changes in SaO_2_, PaO_2_, and PaCO_2_ were minimal, and similar between protocols.

### Effect of hypoxia on cerebrovascular responses to LBNP

Baseline MCAv, CVCi, and CCA diameter were comparable between normoxia and hypoxia (Table 1). Progressive LBNP evoked similar reductions in MCAv during both protocols (Fig. 4). LBNP increased CVCi during hypoxia (*P* = 0.04) versus normoxia. CCA diameter remained constant and was not different between protocols. The slope of the amalgamated arterial pressure‐mean cerebral blood velocity stimulus response curve was lower during hypoxia than normoxia (Fig. 5).

There were no differences in MAP and MCAv power spectral densities or transfer function measures at baseline (Table 1). LBNP increased MAP LF power spectral density during hypoxia versus normoxia (*P* = 0.02) (Fig. 6). Paired *t*‐tests showed that MAP LF power spectral density was significantly different during the final LBNP stage (*P* = 0.01). Similarly, MCAv LF power spectral density increased during hypoxia versus normoxia (*P* < 0.02) with a difference between LBNP‐induced changes during the final LBNP stage (*P* = 0.04). Transfer function analysis revealed that MAP‐MCAv LF transfer function gain increased during LBNP under hypoxic conditions versus normoxic conditions (*P* < 0.01; Fig. 7).

### Hemodynamic, respiratory, and cerebrovascular values prior to presyncope

Hemodynamic, respiratory, and cerebrovascular values were similar in the minute prior to presyncope and discontinuation of LBNP during hypoxia versus normoxia (Table 2).

## Discussion

We compared the cerebral blood flow velocity responses to LBNP during hypoxia and normoxia. The main findings from this investigation are (1) exposure to hypoxia reduced the number of subjects that completed the LBNP protocol and resulted in greater reductions in MAP; (2) the cerebral blood flow velocity responses showed a compensatory increase in conductance during hypoxia as indicated by a higher CVCi and lower slope of the amalgamated arterial pressure‐cerebral blood velocity stimulus response curve; and (3) LF MAP and LF MCAv spectral power density, as well as LF MAP‐mean MCAv transfer function gain increased more during hypoxia.

The decreased tolerance to central hypovolemia with hypoxemia we found in the current study is consistent with previous reports studying head‐up tilt (Malhotra and Murthy 1977; Fulco et al. 1985; Sagawa et al. 1993; Rickards and Newman 2002; Blaber et al. 2003; Halliwill and Minson 2005) and LBNP (Heistad and Wheeler 1970; Rowell and Blackmon 1989; Rowell 1993) with exposure to acute hypoxia. Most of these investigations concluded (Heistad and Wheeler 1970; Fulco et al. 1985; Rowell and Blackmon 1989; Rowell 1993; Sagawa et al. 1993; Blaber et al. 2003; Halliwill and Minson 2005) that hypoxic syncope results from the vasodilator effects of hypoxia on the cardiovascular system (Weisbrod et al. 2001), which is consistent with the lower TPR during hypoxia we encountered at baseline. From the aforementioned studies only Blaber et al. (2003) measured cerebral blood flow responses in addition to cardiovascular and respiratory responses. Consistent with our findings, Blaber et al. (2003) reported similar cerebral blood flows during after one minute of head‐up tilt under normobaric (375 m) and hypobaric conditions (simulated altitude of 3660 m). Additionally, they analyzed normobaric and hypobaric baseline (supine) physiological predictors of time to presyncope during head up tilt at 3660 m. One of the physiological predictors they assessed was CO_2_ reactivity of cerebral blood flow measured during a hyperventilatory maneuver. The multiple regression analysis between normobaric values and time to presyncope at head up tilt at altitude demonstrated that baseline MCAv, CO_2_, respiration rate, and low frequency to high frequency heart rate variability power ratio were predictive of hypoxic head‐up tilt time to presyncope. Thus, hypoxic syncope during head up tilt appears to be related to insufficient cerebral perfusion owing to cerebral vasoconstriction as a result of hypoxic hyperventilation‐induced hypocapnia. We did not find an increase in ventilation or a reduction in PaCO_2_ during LBNP with or without hypoxemia, which is consistent with other studies (Rickards and Newman 2002; Halliwill and Minson 2005). This difference may be explained by the fact that Blaber et al. only measured and reported cerebral hemodynamics a couple minutes into the head up tilt at altitude, and based their conclusions on the supine normobaric baseline predictors of hypobaric head up tilt time to syncope. Other contributing factors to potential differences in ventilatory responses between our study and Blaber et al. (2003) may be differences in ventilatory responses to head up tilt versus LBNP (Bronzwaer et al. 2017), the exposure to relatively modest poikilocapnic hypoxia in the present study, a difference in responses to normobaric versus hypobaric hypoxia (Savourey et al. 2003; Richard and Koehle 2012), the total length of exposure to hypoxia, and/or the gradient between PaCO_2_ and end tidal CO_2_ (Liu et al. 1992). Furthermore, hypoxemia might not produce a pronounced ventilatory response in some individuals (Halliwill and Minson 2005). Therefore, chemoreflex control of ventilation and hemodynamics might be low in these individuals which could predispose them to hypoxic syncope (Halliwill and Minson 2005).

Contrary to our hypothesis, we observed similar cerebral blood flow velocities throughout both protocols, despite a greater decrease in perfusion pressure during the hypoxia protocol. Exposure to acute hypoxia reduced the PaO_2_ to ~50 mmHg, which was accompanied by cerebral vasodilation during LBNP as evidenced by the increased CVCi and improved arterial pressure‐cerebral blood flow relationship during LBNP with hypoxia versus normoxia. These findings suggest adequate static cerebral vascular control occurs during progressive central hypovolemia and hypoxemia, until MAP is decreased to a threshold that adequate cerebral oxygenation can no longer be maintained. We also assessed the arterial pressure‐cerebral blood flow velocity relationship via transfer function analysis, which has been interpreted as an index of dynamic cerebral vascular control (Zhang et al. 1998a). We found greater LF MAP and LF MCAv spectral power density and greater LF transfer function gain during hypoxia versus normoxia. We propose that the observed increase in LF MAP spectral power density, LF MCAv spectral power density, and LF transfer function gain during hypoxemia without marked hypocapnia represents an appropriate compensatory mechanism to maintain cerebral blood flow during periods of low MAP. Similarly, Blaber et al. (2003) reported an attenuated increase in MCAv complexity (measured by fractal component *β* of MCAv) during hypoxic head‐up tilt, which was interpreted as impaired dynamic cerebrovascular control, consistent with the traditional explanation that simultaneous increases in oscillations in MAP and MCAv represent attenuated cerebrovascular control (Zhang et al. 1998b). However, more recently Rickards and Tzeng (2014) reviewed the role of oscillations in MAP and MCAv during hemorrhage and concluded that increased variability may protect cerebral blood flow during hypovolemia. The underlying mechanism may be that less energy is required to maintain forward flow if the flow is pulsatile versus continuous. This is consistent with observations of increased pulsatile cerebral blood flow during LBNP in healthy humans (Thomas et al. 2009; Rickards et al. 2011), in hemorrhaging sheep (Lewis et al. 1999), and in patients with head injury (Czosnyka et al. 1994).

The practical relevance of the changes in cerebral conductance and flow variability during simulated hemorrhage with acute hypoxemia may be that these mechanisms help to maintain cerebral blood flow despite lower MAP during injuries sustained at altitude or chest wall traumas. Our findings render it unlikely that impaired cerebrovascular control contributes to the lower tolerance to central hypovolemia during hypoxia, contrary to what has been suggested by others (Nicholas et al. 1992; Blaber et al. 2003). However, while maintenance of cerebral blood flow is crucial for the delivery of oxygen to the cerebral tissues, the ability to extract and utilize this oxygen may be of even greater importance in terms of tolerance to central hypovolemia. This issue has been highlighted by a number of studies demonstrating that protection of absolute cerebral blood flow does not necessarily provide insight into tolerance to central hypovolemia (Rickards et al. 2011; Jeong et al. 2012; Lucas et al. 2013; Lewis et al. 2014; Kay and Rickards 2016). Cerebral oxygenation, oxygen extraction, and cerebral oxygen metabolism measurements would be interesting additions to comparisons of LBNP‐induced changes during normoxia and hypoxia to address this issue.

### Methodological considerations

Several methodological considerations pertain to our study. First, cerebral blood flow velocity measurements within the MCA are equivalent to flow as long as the caliber of the MCA does not change over the course of the intervention. If MCA diameter was increased during hypoxia, as has been demonstrated at very low PaO_2_ values (Wilson et al. 2011), our measurements may have systematically underestimated blood flow during hypoxia. However, consistent with a study by Ogoh et al. (2014) that explored similar levels of hypoxemia, we found no significant differences in CCA diameter within or between our protocols, suggesting that the hypoxic stimulus was not severe enough to elicit dilatation in the major cerebral arteries. Second, we did not find an increase in ventilation or a reduction in PaCO_2_ during LBNP with or without hypoxemia. Thus, our findings mainly pertain to cerebral blood flow velocity regulation during progressive central hypovolemia without marked hypocapnia. Third, the spectral measures were obtained from the last 3 min of data, which yields a relatively low amount of cycles for analysis in the LF range. We chose this approach to allow 2 min for stabilization of physiological variables at each LBNP stage (Rowell 1993). Previous studies have reported on spectral measures using 3 min of data as well (Rickards et al. 2011, 2015a,b). Fourth, we compared the absolute changes during LBNP versus baseline and we excluded data from LBNP stages at which subjects became presyncopal. We chose this approach to compare the compensatory phase of LBNP exposure between normoxic and hypoxic conditions, since it has been suggested in the literature that LBNP with hypoxemia is associated with impaired cerebrovascular compensation. The actual onset of presyncope is however much more likely dependent on absolute perfusion pressure and cerebral blood flow (Rowell 1993). Moreover, not all subjects reached the same end point during both protocols and within subject endpoints also change between protocols. These different concepts are a returning challenge in the analysis of LBNP studies. To mitigate these issues we included data from both the “survivors” of the LBNP protocols and from those who were more susceptible to LBNP with hypoxemia in a mixed linear models analysis. Estimates of fixed effects in mixed linear models are relatively reliable with small sample sizes (Bell et al. 2010), but future larger scale studies should be performed to validate our findings. In addition to the mixed linear models analysis we compared the absolute hemodynamic, respiratory, and cerebrovascular values during the last minute before presyncope during both protocols and found similar hemodynamic, respiratory, and cerebrovascular values during hypoxia versus nomoxia. Combined with the lower peripheral resistance we found during the hypoxic baseline, and the adequate cerebrovascular response during the compensatory phase of LBNP these data suggest that lower tolerance to LBNP during hypoxemia is not a result of impaired cerebral blood flow control during LBNP with hypoxemia, but more likely is secondary to lower perfusion pressure as a result of impaired systemic peripheral vasoconstriction. Fifth, we did not test women, but would hypothesize differential responses between men and women because women typically have a lower orthostatic stress tolerance than men (Franke et al. 2003), which may affect hemodynamic (Gotshall et al. 1991) and cerebrovascular (Deegan et al. 2010) responses to orthostatic stress. Sex‐specific data on the hemodynamic and cerebrovascular responses to LBNP during normoxia and hypoxia would be a valuable addition to the results described in the current report.

## Conclusions

The findings from this study indicate that acute hypoxemia during simulated hemorrhage induces changes in cerebral conductance and flow variability that may help to maintain cerebral blood flow despite lower MAP. It is unlikely that impaired cerebrovascular control contributes to the lower tolerance to central hypovolemia during hypoxia.

## Conflict of Interest

No conflicts of interest.

## Disclaimer

The opinions or assertions contained herein are the private views of the authors and are not to be construed as official or as reflecting the views of the US Department of the Army or the US Department of Defense.
